# Targeted near-infrared imaging utilizing a cathepsin-activated fluorophore for the intraoperative detection of canine insulinoma

**DOI:** 10.1371/journal.pone.0343299

**Published:** 2026-02-23

**Authors:** Maureen A. Griffin, Enrico Radaelli, Charles-Antoine Assenmacher, Sunil Singhal, Robert E. Roses, Brian K. Flesner, Darko Stefanovski, Nimar Gill, Jennifer L. Huck, Heather Scavello, Jillian Verrelle, Kristen Farrell, David E. Holt

**Affiliations:** 1 Department of Clinical Sciences and Advanced Medicine, University of Pennsylvania School of Veterinary Medicine, Philadelphia, Pennsylvania, United States of America; 2 Department of Pathobiology, Penn Vet Comparative Pathology Core, University of Pennsylvania School of Veterinary Medicine, Philadelphia, Pennsylvania, United States of America; 3 Department of Surgery, University of Pennsylvania School of Medicine, Philadelphia, Pennsylvania, United States of America; 4 Cancer Biology and Experimental Therapeutics Laboratory, Colorado State University College of Veterinary Medicine and Biomedical Sciences, Fort Collins, Colorado, United States of America; Colorado State University, UNITED STATES OF AMERICA

## Abstract

**Objectives:**

The aim of this study was to evaluate the use of cathepsin-activated intraoperative near-infrared (NIR) imaging to detect insulinomas in dogs, a spontaneous large animal model for human disease.

**Materials and methods:**

A prospective, pilot clinical trial was performed on dogs with naturally occurring insulinomas undergoing exploratory laparotomy. Each dog underwent routine preoperative diagnostic assessment, and a cathepsin-activated fluorophore (VGT-309) was administered intravenously 1–2 days preoperatively. All intraoperative findings with visible light and NIR imaging were recorded and mean NIR fluorescence intensity of tumors and grossly normal pancreas were quantified. Excision of any identified primary tumor and suspected metastatic lesions was performed. All excised tissues underwent histologic evaluation and immunohistochemistry (IHC) for cathepsin B expression. Descriptive statistics were calculated, and differential fluorescence intensity and cathepsin B expression between the pancreatic mass and adjacent grossly normal pancreatic tissue were assessed for statistical significance via paired t tests with *p* < 0.05 used for significance.

**Results:**

Six dogs were enrolled. No adverse events occurred secondary to administration of the imaging agent. In situ, insulinomas had significantly greater mean fluorescence intensities than the surrounding pancreas, and the median tumor to background ratio was 1.906 (range 1.286–2.556). One dog had an occult pancreatic mass that was identified intraoperatively with NIR guidance. Background fluorescence of liver and lymph nodes was observed in all cases, and one dog was diagnosed with nodal and hepatic metastasis. Histologic tumor margins correlated with margins of NIR fluorescence. Cathepsin B expression was determined to be significantly greater in the pancreatic tumor compared to adjacent non-neoplastic pancreas via IHC, and there was no overlap in the range of median IHC-positive proportion values for these tissues. However, there was overlap in the range of IHC-positive proportion values for neoplastic pancreatic samples and lymph node and liver tissues.

**Clinical significance:**

The findings of this pilot study support further investigation of cathepsin-activated NIR imaging to enhance intraoperative canine insulinoma localization and margin evaluation. Future studies are needed to further characterize and optimize the utility of targeted NIR imaging, particularly to identify metastatic lesions, for canine insulinoma, which may serve as an effective translational model for humans with pancreatic neuroendocrine tumors.

## Introduction

Insulinoma is the most common endocrine pancreatic tumor in dogs and humans [1–3]. Surgical excision of the tumor and metastatic lesions is generally indicated for neoplastic disease control and glycemic regulation, and surgical management results in better outcomes than medical management alone for dogs with insulinoma [2,4]. Pre- and intra-operative tumor detection is therefore vital to optimize outcomes. However, pre- and intra-operative detection of insulinomas and other pancreatic neuroendocrine tumors can be challenging in both canine and human patients. Abdominal ultrasonography accurately identifies and localizes pancreatic masses in less than 50% of dogs with insulinoma [5]. Magnetic resonance imaging (MRI) and contrast-enhanced computed tomography (CT) are unsuccessful in localizing human insulinomas preoperatively in 25–35% of patients [6]. In a canine insulinoma study, contrast-enhanced CT identified a lesion in 96% of cases, but accuracy relative to localization of an insulinoma lesion was only 52% with 33% of dogs having CT-based location errors that were considered major [7]. Dual-phase CT angiography has shown promise with hyperattenuation in 82% and deformation of pancreatic shape in 79% of dogs with insulinoma, although histology was only performed in 60% of cases to determine accuracy of these findings [8]. A recent study demonstrated that the late arterial phase of a triple arterial phase CT angiography protocol resulted in the greatest number of nodules and enhancement scores, though the majority of cases (67%) also did not have corresponding histology of the lesions to confirm correlation with neoplasia [9]. MRI for insulinoma detection has been reported in only four canine patients; all dogs displayed imaging changes associated with the insulinoma [10]. Positron emission tomography (PET)/CT has been used in humans to localize insulinomas by targeting glucagon-like peptide-1 receptor (GLP-1R), but only one-third of malignant tumors express GLP-1R [11,12]. However, malignant insulinomas typically express somatostatin receptors, and somatostatin receptor-based analogues, such as ^68^Gallium-DOTA-(Tyr3)-octreotate, have been used successfully as targets in PET/CT imaging to localize both benign and malignant insulinoma lesions in humans [13–15]. PET/CT with ^18^F-fluorodeoxyglucose has recently been described as a preoperative imaging modality in dogs with insulinoma, though avidity of the insulinoma lesions was found to be inconsistent and availability of this equipment is generally limited in veterinary medicine [16]. Agreement between pre- and intra-operative findings have been inconsistent in dogs with insulinoma [1,5,7].

Surgical exploration is indicated for excision of primary and metastatic insulinomas when feasible. The majority of dogs with insulinoma have a single primary pancreatic nodule identified intraoperatively; however, historical reports document multiple pancreatic masses in 8–15% of dogs and pancreatic insulinoma lesions that are not palpable at the time of surgery in up to 20% of dogs [1,17–19]. Selective arterial calcium stimulation testing with hepatic venous sampling has been performed to localize an insulinoma to within an arterial territory in human patients without successful preoperative localization, though it does not localize the tumor itself within the pancreatic tissue and is an invasive procedure with risk for complications [11,20]. In dogs, intravenous (IV) administration of methylene blue has been described to aid identification of neoplastic islet cells if no tumor is grossly visible or palpable; however, maximal staining is delayed and adverse effects associated with methylene blue administration such as Heinz body anemia and renal injury are possible [2,19]. As a result, this technique is not widely used.

The fundamental challenge of intraoperative identification of tumors, their margins, and regional metastatic disease has prompted investigations into real-time intraoperative molecular imaging. Sentinel lymph node mapping via intraoperative near-infrared (NIR) imaging using intratumoral injection of indocyanine green has been reported in a dog with an insulinoma to guide targeted extirpation and evaluation of lymph nodes at greatest risk of metastatic disease [21]. Alternatively, the use of systemically injected NIR contrast agents is a relatively novel method for intraoperative disease localization that is being investigated in numerous malignancies to localize both primary tumors and metastatic lesions [22]. Multiple specific, targeted NIR fluorescent probes have been developed for NIR tumor localization, allowing visualization of the tumor and/or tumor microenvironment during surgery using sensitive charge-coupled device cameras to optimize tumor detection, delineate tumor margins, and preserve normal structures [23–27]. VGT-309 (Vergent Bioscience, Minneapolis, MN) is a cathepsin-activated NIR fluorophore that is safe in normal dogs and has demonstrated enhanced NIR fluorescence of primary lung tumors in a translational study in preclinical mouse models, dogs, and humans [28]. Cathepsins are lysosomal proteases that may be secreted by cancer cells at greater levels than normal cells and are activated to alter the tumor microenvironment for growth and metastasis [29,30]. Importantly, human insulinoma cells contain cathepsins B, H, and L [31,32]. Cathepsin B and L expression is upregulated in human insulinoma compared to normal pancreatic tissue controls, and cathepsin B has been shown to play a role in metabolism of human insulinoma cells [32,33]. These findings suggest that the use of cathepsin-activated NIR intraoperative imaging may be beneficial in localizing insulinomas.

Evaluation of targeted NIR imaging techniques in dogs with insulinoma may prove useful in enhancing outcomes in dogs with naturally occurring disease and their human counterparts. Studies on client-owned animals with spontaneously occurring neoplastic disease serve as effective large animal, translational models of human neoplasia. Such studies have previously demonstrated that NIR imaging can accurately localize and detect tumor margins and residual local disease in canine primary lung tumors, sarcomas, and mammary tumors [28,34–37]. Further investigation into effective strategies to accurately localize disease burden intraoperatively in dogs with insulinoma, utilizing intraoperative cathepsin-activated fluorophores with NIR imaging, may serve as an effective model to drive further research of these targeted imaging agents and NIR technology in their human counterparts [38]. In this study, we aimed to evaluate the use of a cathepsin-activated fluorophore and intraoperative NIR imaging to detect insulinomas in dogs, a spontaneous large animal model for the corresponding human disease [38]. The objectives of the study were to: i) determine whether a cathepsin-activated fluorophore and NIR imaging reliably detects canine insulinoma; and ii) confirm that cathepsin-activated NIR fluorescence is associated with cathepsin expression.

## Methods

A prospective clinical trial was performed at the University of Pennsylvania Matthew J. Ryan Veterinary Hospital. Inclusion criteria were as follows: client-owned dogs with naturally occurring insulinomas diagnosed via hypoglycemia with concurrent inappropriately normal or elevated serum insulin levels that had not received prior surgery, radiation therapy, or chemotherapy treatment. All owners elected surgical treatment via exploratory laparotomy prior to inclusion in the study. The study protocol was approved by the University’s Institutional Animal Care and Use Committee and the School’s Privately Owned Animal Protocol review committee. Written informed client consent was obtained for each patient prior to enrollment. Before enrollment, each dog was required to have pre-anesthetic clinical laboratory testing consisting of complete blood count and biochemistry panel, and standard of care preoperative imaging (including thoracic radiography and/or CT, and abdominal ultrasonography and/or CT angiography) was offered to all clients. Signalment, history, clinical signs, physical examination findings, all diagnostic testing results, and any preoperative treatments were recorded.

Prior to surgery, dogs were administered VGT-309 0.2 mg/kg IV. During and following the injection, dogs were monitored for any adverse events including signs of nausea and cardiorespiratory abnormalities. A standard of care exploratory laparotomy was subsequently performed under general anesthesia. Anesthetic protocols were performed at the discretion of the attending anesthesiologist. All intraoperative findings with visible light were documented. NIR imaging guidance was also used to image and evaluate lesions consistent with primary insulinomas and metastatic disease within the abdomen. Imaging was performed with a VisionSense Iridium NIR imaging system (Medtronic, Minneapolis, MN), and all findings were recorded. This high definition, dual band (visible light and NIR) camera system utilized an 805-nm excitation source, and NIR fluorescence was detected with a bandpass filter ranging from 825–850 nm. A free-standing exoscope was used with a field of view of approximately 19 × 14 cm and a working distance of approximately 40 cm. Variable gain was utilized with settings optimized by the imaging system. When NIR imaging was performed, ambient light was removed by covering all windows with shades and turning off all overhead and surgical lights. Following complete abdominal exploration, standard of care surgery for insulinoma was performed with excision of any identified primary tumor and excision or sampling of any potential metastatic lesions via routine surgical techniques. Postoperatively, all dogs were treated and monitored according to standard of care.

Following excision, primary tumors were imaged via NIR. Margins of fluorescence were marked with suture at sites of transition in fluorescence intensity within the excised tissues (where adequate residual pancreas of reduced fluorescence was available). Margins of excision were marked with ink. Histopathological evaluation of all excised tissues and their margins was routinely performed, and results were documented. When obtained, sutured fluorescent margins of the primary tumor were compared to histopathological margins of disease. Following validation of immunohistochemistry (IHC) antibodies for cathepsins in canine tissues, excised tissues including the pancreatic mass and adjacent non-neoplastic pancreatic tissue (excised with the tumor at the grossly normal margin) were quantitatively assessed and compared for cathepsin expression by IHC. The cathepsin antibody, rabbit monoclonal recombinant anti-cathepsin B [D1C7Y, 31718] (Cell Signaling Technology, Danvers, MA), was evaluated for validation in canine tissue. The IHC protocol is described in S1 Appendix. The cathepsin B antibody resulted in specific labeling of canine pancreatic islets (positive control) with no labeling of irrelevant isotype-matched rabbit monoclonal antibody (negative control). In addition, the cathepsin B antibody was validated via western blot, results of which demonstrated appropriate specificity and cross-reactivity. The western blot protocol is described in S2 Appendix, and Fig S3 in S1 File demonstrates the western blot results. This antibody was used for IHC evaluation of excised pancreatic, lymph node, and liver tissue samples from the included dogs. IHC slides were scanned using the Aperio Versa 200 scanner (Leica Biosystems, Buffalo Grove, IL) and five square sections of 0.25 mm^2^ were randomly selected from tumor and non-tumor tissues at low magnification (0.9x). Any areas with artifact (such as edge effect and excessively dark intensity of staining) were avoided in the random selection regions. The number of IHC positive pixels was quantified within these regions using a positive pixel count algorithm on the Aperio ImageScope software. Three positive staining intensity thresholds were selected and other parameters were tuned to include the entire range of positive pixels while excluding background staining (Table S1 in S1 File). The proportion of IHC positivity was defined as the number of positive pixels divided by the total number of pixels within the given area. The IHC-positivity ratio (IHCr) was calculated by dividing the IHC positivity of the pancreatic tumor to that of the adjacent non-neoplastic pancreas. Mean fluorescence intensity was quantified for all imaged tissues using ImageJ (U.S. National Institutes of Health, Bethesda, MD), with outlines drawn around gross tumor and grossly normal tissues immediately adjacent to the tumor. Mean fluorescence intensity assessment was performed in triplicate for each tissue type and results were averaged. Tumor to background signal ratios (TBR) were calculated by dividing the triplicate mean fluorescence intensity of the tumor region of interest by the triplicate mean fluorescence intensity of the adjacent grossly normal pancreas.

### Statistical methods

All analyses were conducted with Stata 18MP, StataCorp, College Station TX, with two-sided tests of hypotheses and a *p*-value < 0.05 as the criterion for statistical significance. Descriptive analyses included computation medians and ranges of continuous variables and tabulation of categorical variables. Tests of normal distribution (Shapiro-Wilk test) were performed to determine extent of skewness of continuous variables. Frequency counts and percentages were used for summarizing categorical variables.

Paired t tests were used to compare both in situ fluorescence intensity and IHC cathepsin B expression of the pancreatic tumor tissues relative to adjacent grossly normal pancreatic tissues for all dogs, with alpha set at 0.05. For all fluorescence data, the triplicate measurement average was used for analysis. For dogs in which multiple representative IHC slides for given tissue samples were available, the average proportion of IHC positivity was used for this calculation. Results were reported as means and mean differences with their respective 95% confidence interval (95% CI) and the *p*-value of the pairwise comparison.

## Results

### Demographic data and diagnostic assessment

A single dog (Dog 1) that met all study inclusion criteria underwent the trial protocol prior to initiation of the clinical trial as a proof of concept. Subsequently, five additional dogs were enrolled, such that six dogs were included in the study data. The following breeds were represented: Chihuahua, Bernese Mountain Dog, Yorkshire Terrier, Labrador Retriever, Portuguese Water Dog, and mixed. All dogs were spayed females. The median age was 6.9 years (range 4.3–9.5). The median body weight was 23.0 kg (range 3.9–50.0). Reported clinical signs of insulinoma included seizures (1), ataxia (2), tremors (2), atypical behavior (1), and collapse (1); hypoglycemia was detected incidentally with no clinical signs reported in three dogs.

Preoperative abdominal imaging was performed in all dogs and consisted of ultrasonography by a board-certified radiologist in all six dogs and CT angiography in four dogs. For dogs that underwent CT angiography, a pre-contrast CT was performed followed by dual phase arterial angiogram (with early and late arterial phases) and delayed/venous phase. Results of the abdominal ultrasound and CT scans are recorded for each dog in Table 1.

**Table 1:** Pertinent results of abdominal ultrasonography (US), abdominal CT angiography, surgical findings (including both visible light and NIR), and surgical procedures with World Health Organization TNM stage based on histology results for each dog. [17] Pancreatic mass locations: L (left lobe with further location not specified), LD (distal left lobe), LP (proximal left lobe), B (body), R (right lobe with further location not specified), RP (proximal right lobe), RD (distal right lobe).

Dog ID and Signalment	Pancreatic Mass Findings	Other Pertinent Findings	Surgical Excisions Performed and TNM Stage
1 (6 year old FS Chihuahua)	**US:** 1.2 x 1.4 cm mass in B. **CT:** 2.2 x 1.9 x 1.2 cm mass in L. **Surgery:** 2 x 1 cm mass in LD, greater fluorescence identified relative to surrounding pancreas.	**US:** None. **CT:** 5 mm lesion in RP on one reformat only (suspected artifact). Splenic lymph node mildly enlarged (4–5 mm) and strongly enhancing. **Surgery:** Fluorescence of lymph nodes, liver, and GI tract diffusely (liver most intense, GI least intense).	**Surgical Procedures:** Partial pancreatectomy **Stage:** T1N0M0
2 (4 year old FS Bernese Mountain Dog)	**US:** No mass. **CT:** 1.1 x 1.1. x 1.4 cm mass in LD. **Surgery:** 1 x 1.5 cm mass in LD, greater fluorescence identified relative to surrounding pancreas.	**US:** None. **CT:** Enlarged splenic lymph node (10.5 mm). **Surgery:** Prominent splenic lymph node. Fluorescence of lymph nodes, liver, and GI tract diffusely (liver most intense, GI least intense).	**Surgical Procedures:** Partial pancreatectomy, lymph node extirpation (colonic and splenic), liver biopsy **Stage:** T1N0M0
3 (7 year old FS Yorkshire Terrier)	**US:** 3.5 mm nodule in R. **CT:** 6 mm nodule in RD. **Surgery:** 1 cm mass in RD, greater fluorescence identified relative to surrounding pancreas.	**US:** Nodules (2.4 mm, 2.1 mm) in region of splenic lymph nodes. **CT:** Pancreaticoduodenal lymph node enlarged (6.1 mm). **Surgery:** Fluorescence of lymph nodes, liver, and GI tract diffusely (liver most intense, GI least intense). Focal pale lesion in left lateral liver (2 mm), homogeneous in fluorescence intensity compared to adjacent grossly normal liver.	**Surgical Procedures:** Partial pancreatectomy, liver biopsy. **Stage:** T1N0M0.
4 (8 year old FS Labrador)	**US:** 2 x 2.7 cm mass in B/LP. **CT:** Not performed. **Surgery:** Small lesion (several mm) in RP identified via enhanced fluorescence relative to surrounding pancreas – not identified upon initial visual inspection or palpation of pancreas.	**US:** Mild hepatic heterogeneity. **CT:** Not performed. **Surgery:** 3 cm mid pancreaticoduodenal lymph node. Cranial pancreaticoduodenal lymph node firm and 1 cm. Fluorescence of lymph nodes, liver, and GI tract diffusely (liver most intense, GI least intense). Multiple (~20) pale pinpoint lesions throughout liver, homogeneous in fluorescence intensity compared to adjacent grossly normal liver.	**Surgical Procedures:** Partial pancreatectomy, lymph node (large mid pancreaticoduodenal, smaller cranial pancreaticoduodenal) extirpation, liver biopsies. **Stage:** T1N1M1
5 (6 year old FS mixed breed)	**US:** 1.6 x 1.4 cm mass in L. **CT:** Not performed. **Surgery:** 1.5 x 2 cm mass in LD, greater fluorescence identified relative to surrounding pancreas.	**US:** None. **CT:** Not performed. **Surgery:** Fluorescence of lymph nodes, liver, and GI tract diffusely (liver most intense, GI least intense).	**Surgical Procedures:** Partial pancreatectomy, liver biopsy **Stage:** T1N0M0
6 (10 year old FS Portuguese Water Dog)	**US:** 2.4 x 1.2 cm mass in B. **CT:** 2.5 x 1.2 cm mass in B/RP. **Surgery:** 2.5 cm mass in LP/B; LD atrophied and pale, greater fluorescence identified relative to surrounding pancreas.	**US:** Mild hepatic lymphadenopathy (5.5 mm, 8 mm). **CT:** Lymphadenopathy of hepatic (up to 9 mm) and additional regional lymph node. **Surgery:** Pancreatic lymph node closely associated with pancreatic mass. Splenic lymph node firm, dark, 1 cm. Fluorescence of lymph nodes (including pancreatic and splenic), liver, and GI tract diffusely (liver most intense, GI least intense). Liver enlarged and firm with multifocal pale pink nodules (~5 mm) throughout lobes (1–3 per lobe). When gain decreased to 4%, liver nodule intensity appeared greater than surrounding liver; at higher gain, liver appeared homogeneously fluorescent. Omental nodule 0.5 x 1 cm with no fluorescence detected.	**Surgical Procedures:** Partial pancreatectomy, lymph node (pancreatic and splenic) extirpation, liver biopsies, omental nodule extirpation. **Stage:** T1N0M0

Preoperative thoracic imaging was performed in all dogs and consisted of 3-view thoracic radiographs in 5/6 dogs. No pulmonary metastasis was detected radiographically in any case. The only dog (Dog 6) that did not undergo thoracic radiographs underwent thoracic CT for staging. Thoracic CT revealed an irregular, 5.8 mm nodule within the left cranial lung lobe with both benign and malignant etiologies considered.

Preoperatively, all dogs were medically managed for their insulinoma with diet modification (frequent small feedings of a diet high in protein and complex carbohydrates) and limited excitement and activity. Prednisone was administered in two dogs (Dogs 1 and 5) preoperatively: 0.5–0.9 mg/kg/day for 3–4 weeks. The dog that presented with seizures (Dog 1) also received phenobarbital and levetiracetam.

### Surgical, NIR, and histologic findings

VGT-309 (0.2 mg/kg IV) was administered over approximately 15 minutes 18–24 hours preoperatively in 5/6 dogs (Dogs 1–5) and 48 hours preoperatively in 1/6 dog (Dog 6). No adverse events associated with VGT-309 administration were documented. Findings from abdominal exploration under visible light and NIR imaging are documented for each dog in Table 1. A single pancreatic mass was detected and excised via partial pancreatectomy in all six dogs. Lymph nodes were extirpated in 3/6 dogs, and liver biopsies were obtained in 5/6 dogs. The ImageJ fluorescence intensity data for all dogs is provided in Table 2.

**Table 2:** ImageJ analysis results of fluorescence intensity and IHC results of relevant excised tissues for each dog. The ratio of IHC positivity (IHCr) between pancreatic mass and adjacent non-neoplastic pancreas has also been provided for each dog. When multiple IHC slides were available for a given tissue type, the average positivity for the tissue type in the dog was recorded. Histologic evidence of metastasis within the sample is denoted (M). All findings (including fluorescence intensity and standard deviation) are averages of measurements performed in triplicate for each tissue type. Gain settings are reported for each recording. All provided measurements were obtained from in situ images unless otherwise noted.

Dog	Mean fluorescence intensity (pixels) of tissues with histologic samples and tumor to background signal ratios (TBR) of the pancreatic mass relative to adjacent grossly normal pancreas	IHC results: positivity and ratio between pancreatic mass and adjacent pancreas (IHCr)
1	**Pancreatic mass:** 177.114 (SD 9.691, gain 10%). **Adjacent grossly normal pancreas:** 122.168 (SD 18.406, gain 10%) **TBR:** 1.450	**Pancreatic mass:** 0.654. **Adjacent grossly normal pancreas:** 0.064. **IHCr:** 10.219
2	**Pancreatic mass:** 146.802 (SD 12.851, gain 23%). **Adjacent grossly normal pancreas:** 114.185 (SD 15.244, gain 23%). **Colonic lymph node:** 206.849 (SD 13.330, gain 23%). **Splenic lymph node:** 208.948 (SD 10.212, gain 21%). **Liver:** 145.561 (SD 24.623, gain 11%). **TBR:** 1.286	**Pancreatic mass:** 0.595. **Adjacent grossly normal pancreas:** 0.118. **Lymph nodes** – cortex: 0.134; medulla: 0.417. **Liver:** 0.659. **IHCr:** 5.042
3	**Pancreatic mass:** 157.471 (SD 17.133, gain 34%). **Adjacent grossly normal pancreas:** 61.615 (SD 13.170, gain 34%). **Liver:** 146.097 (SD 36.639, gain 14%). **TBR:** 2.556	**Pancreatic mass:** 0.488. **Adjacent grossly normal pancreas:** 0.057. **Liver:** 0.403. **IHCr:** 8.561
4	**Pancreatic mass:** 184.094 (SD 23.425, gain 27%). **Adjacent grossly normal pancreas:** 84.172 (SD 19.898, gain 27%). **Large mid pancreaticoduodenal lymph node (M):** 79.315 (SD 12.235, gain 27%). **Smaller cranial pancreaticoduodenal lymph node:** 94.143 (SD 16.815, gain 19%). **Liver (M):** 74.290 (SD 13.235, gain 13%). **TBR:** 2.187	**Pancreatic mass:** 0.749. **Adjacent grossly normal pancreas:** 0.103. **Lymph node (M):** 0.752. **Lymph node** – cortex: 0.471; medulla: 0.560. **Liver (M):** 0.636; normal liver: 0.379. **IHCr:** 7.272
5	**Pancreatic mass:** 112.974 (SD 17.990, gain 49%). **Adjacent grossly normal pancreas:** 58.350 (SD 11.949, gain 49%). **Liver:** 211.712 (SD 21.495, gain 24%). **TBR:** 1.936	**Pancreatic mass:** 0.398. **Adjacent grossly normal pancreas:** 0.105. **Liver:** 0.375. **IHCr:** 3.790
6	**Pancreatic mass and closely associated lymph node:** 118.134 (SD 21.997, gain 24%). **Adjacent atrophied pancreas:** 62.993 (SD 10.907, gain 24%). **Liver nodule:** 139.320 (SD 2.536, gain 4%). **Adjacent grossly normal liver:** 104.616 (SD 20.235, gain 4%). **Omental nodule:** 8.246 (SD 2.445, gain 24%). **Splenic lymph node (ex vivo):** 45.722 (SD 8.158, gain 29%). **TBR:** 1.875	**Pancreatic mass:** 0.739. **Adjacent grossly normal pancreas:** 0.177. **Lymph node** – cortex: 0.323; medulla: 0.605. **Liver:** 0.601. **IHCr:** 4.175

### NIR fluorescence – pancreatic tissues

**Fig 1:**
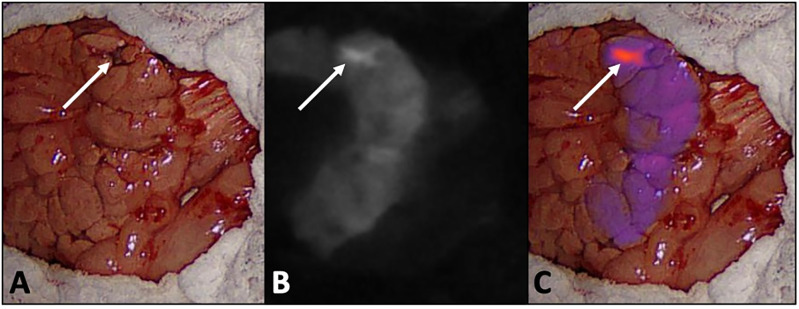
Intraoperative image with visible light alone (A), NIR alone (B), and an overlay of NIR findings on visible light (C) of the right pancreatic lobe from Dog 4 (the same dog with IHC depicted in Fig 4). All tissues aside from the pancreas and mesoduodenum have been covered with laparotomy sponges. This dog had a small pancreatic nodule (denoted by white arrows) that was not definitively identified upon initial visualization or palpation of the pancreas but was identified following application of NIR imaging due to a focal region of enhanced fluorescence intensity relative to the surrounding pancreas.

Insulinomas were visible with NIR imaging intraoperatively and distinguishable from normal pancreas in all cases. In five dogs, the insulinoma was initially identified by gross visualization and palpation. In one dog (Dog 4), a lesion described as a pancreatic mass on preoperative ultrasound was identified as a pancreaticoduodenal lymph node at surgery. No pancreatic mass was identified by visual inspection or palpation of the pancreas. NIR imaging of the pancreas revealed a focal region of enhanced fluorescence intensity in the proximal right pancreatic lobe. A very small (several mm), firm pancreatic nodule was identified in this location. This nodule was subsequently excised and confirmed to be an insulinoma on histologic evaluation. Fig 1 depicts intraoperative images of this lesion via visible light and NIR imaging.

Fluorescence intensity data for pancreatic mass and adjacent grossly normal pancreas groups was normally distributed. Mean fluorescence intensities of the pancreatic masses and adjacent normal pancreatic tissues were 149.432 (95% CI 118.485–180.378) and 83.914 (95% CI 54.349–113.478), respectively. The mean difference in fluorescence intensity between the two groups was 65.518 (95% CI 37.655–93.381). The pancreatic masses had significantly greater NIR fluorescence mean intensity compared to the grossly normal pancreatic tissues adjacent to the mass in all dogs (*p* = 0.0018). The mean TBR was 1.906 (range 1.286–2.556); two dogs had TBRs < 1.5. No additional regions of relatively enhanced pancreatic fluorescence were identified in any case.

### NIR fluorescence – non-pancreatic tissues

**Fig 2:**
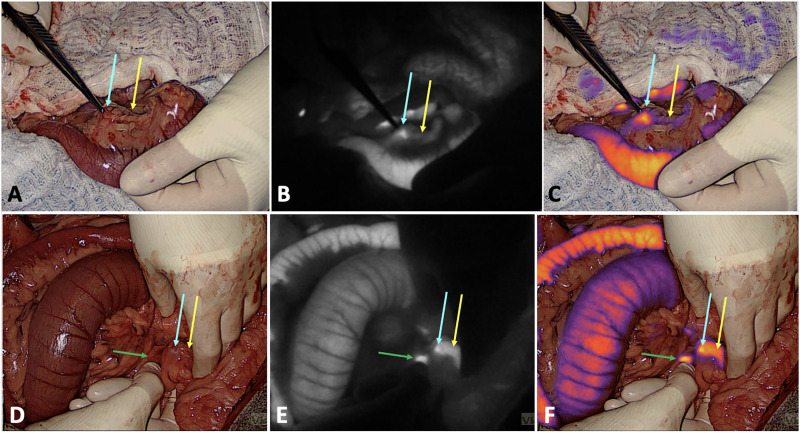
Intraoperative images with visible light alone (A and D), NIR alone (B and E), and an overlay of NIR findings on visible light (C and F) of the pancreatic mass from Dog 3 (A, B, and C) and Dog 2 (D, E, and F), respectively. The blue arrow points to the pancreatic mass, and the yellow arrow points to the adjacent grossly normal pancreas. The green arrow in Dog 2 images points to a colonic lymph node. In images **B, C, E,** and **F,** enhanced fluorescence of the pancreatic tumor relative to grossly normal adjacent pancreas is evident as is generalized background fluorescence of the adjacent gastrointestinal tissues (Dogs 3 and 2) and colonic lymph node (Dog 2). Dogs 3 and 2 had the greatest and lowest TBRs, respectively, such that these images depict representative intraoperative findings of the range of fluorescence discrepancy between pancreatic tumors and the adjacent pancreas in dogs of this study.

Substantial background NIR fluorescence of the liver, lymph nodes, and gastrointestinal tract was observed in all dogs. Fig 2 depicts representative intraoperative images from the dogs with greatest and lowest TBR values (Dogs 3 and 2, respectively), with background fluorescence of the gastrointestinal tract and lymph nodes evident. The background fluorescence intensity was greatest for the liver in all dogs, which displayed diffuse fluorescence. In a single case (Dog 6) with hepatic nodules, one of the nodules appeared to have greater fluorescence intensity relative to the surrounding liver when low gain settings were applied. However, this did not correlate with metastasis on histologic evaluation. One dog (Dog 4) did have histologic evidence of hepatic metastasis in association with multiple pinpoint pale nodules within the liver, and the liver appeared diffusely fluorescent on NIR imaging with no alteration in fluorescence intensity in the regions of the hepatic nodules, even at relatively low gain settings. Moreover, though lymph nodes generally fluoresced in all cases, the intensity of nodal fluorescence relative to the pancreatic mass was variable. In one dog (Dog 4), a metastatic lymph node had a lower mean fluorescence intensity than the pancreatic insulinoma and another non-metastatic lymph node. Non-metastatic lymph nodes in other dogs had greater (Dog 2) and similar (Dog 6) mean fluorescence intensity relative to the insulinoma.

### Pancreatic margins

**Fig 3:**
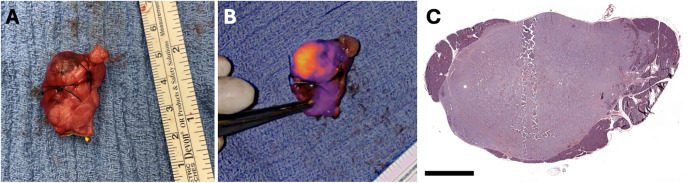
Images of the partial pancreatectomy sample in Dog 5. **A)** Ex vivo image of left partial pancreatectomy in visible light; the margins of excision have been marked with ink (yellow), and the margins of enhanced fluorescence intensity have been marked with suture. **B)** Ex vivo image of left partial pancreatectomy with NIR overlay on visible light, demonstrating the suture placement relative to fluorescence. **C)** Formalin-fixed paraffin embedded slide (0.5x) stained with hematoxylin/eosin of the pancreatic lesion, which has been trimmed through the suture sites peripherally; the scale bar represents 4 mm. Neoplastic cells extend to within less than 1 mm of the capsular connective tissue in the sutured sites. (Sutures were removed prior to sectioning.).

The margins of excision incorporated all gross disease and regions of increased pancreatic fluorescence intensity. Larger gross pancreatic margins were limited by the location of the neoplasm relative to important structures including vasculature and ductal structures, with potential for morbidity with a wider excision. In each of these cases, the margins of fluorescence corresponded closely with histologic margins, and complete histologic excisions were achieved. In two cases (Dogs 5 and 6), the margins of NIR fluorescence were able to be individually marked with suture and evaluated histologically; representative images from Dog 5 are depicted in Fig 3. The sutured margins correlated closely with histologic margins, with the histologic margin within 1 mm of the sutured tissues. No enhanced residual fluorescence was noted when imaging the remaining transected pancreatic margins, which correlated with complete histologic excision in all cases.

### IHC findings

**Fig 4:**
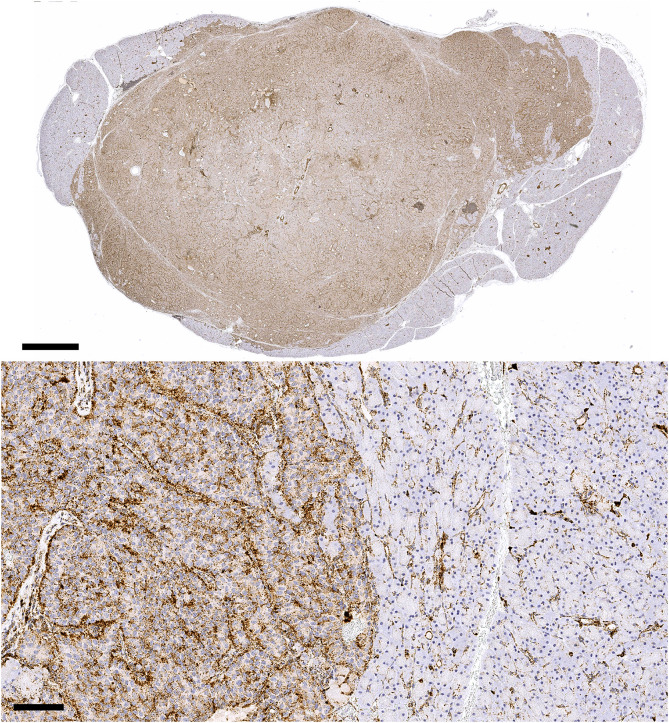
Pancreatic tumor and surrounding non-neoplastic pancreas in Dog 4. Top panel: Formalin-fixed paraffin embedded slide (0.9x) stained with cathepsin B antibody for IHC analysis; the scale bar represents 2 mm. Bottom panel: A close-up image captured at the junction of tumor and normal pancreas shows significantly higher levels of cathepsin B immunoreactivity in the neoplastic tissue; the scale bar represents 100 μm.

Available IHC results for each dog are provided in Table 1, and Fig 4 depicts a representative IHC image of pancreatic tumor and adjacent pancreas for Dog 4; Fig S1 and S2 in S1 File depict representative IHC images of non-metastatic liver and lymph node for Dog 6. The median total area assessed for each tissue type (tumor or non-tumor) on a given slide was 1.165 mm^2^ (range 0.498–1.242). For all available slides, the median IHC-positive proportion for pancreatic insulinoma was 0.551 (range 0.337–0.772) and for non-neoplastic pancreas was 0.082 (range 0.047–0.177). For all available slides, the median IHC-positive proportions for non-metastatic liver tissue, lymph node cortex, and lymph node medulla were 0.403 (range 0.375–0.659), 0.320 (range 0.129–0.471), and 0.553 (range 0.323–0.658), respectively. For the case (Dog 4) with metastatic liver and lymph node samples, the IHC-positive proportion was 0.636 for metastatic liver and 0.752 for metastatic lymph node. The IHC-positive proportion data was normally distributed for pancreatic mass and adjacent grossly normal pancreas groups. Mean IHC-positive proportion of the pancreatic mass was 0.594 (95% CI 0.446–0.742), and mean IHC-positive proportion of the adjacent grossly normal pancreas was 0.104 (95% CI 0.059–0.149). The mean difference in IHC-positive proportion between the two groups was 0.490 (95% CI 0.350–0.629). Cathepsin B expression was significantly greater in the pancreatic tumor compared to surrounding non-neoplastic pancreas via IHC quantitative analysis (*p* < 0.001).

### Outcomes

There were no intraoperative complications in any case. All dogs survived to discharge with a median hospitalization of 4.5 days (range 2–6) postoperatively. Following surgery, 3/6 dogs became euglycemic, 2/6 dogs (Dogs 1 and Dog 5) became hyperglycemic, and 1/6 dog (Dog 3) was hypoglycemic. Dog 3 had an increase in blood glucose intraoperatively (to within normal limits) without dextrose supplementation following pancreatic mass excision. Postoperatively, the dog was initially hypoglycemic but became euglycemic without supplementation or glucocorticoid administration at the time of discharge. The dog was started on prednisone (0.5 mg/kg/day PO) at the time of recheck 3 weeks postoperatively due to recurrent hypoglycemia.

Postoperatively, 4/6 dogs developed evidence of progressive disease based on laboratory or imaging results at a median of 187 days (range 93–383) following surgery. One dog (Dog 4) was diagnosed with stage 3 disease at the time of surgery and had no follow-up imaging. Two dogs (Dog 1 and Dog 3) had evidence of lymphadenopathy and no overt pancreatic lesions on follow-up abdominal imaging, and one dog (Dog 5) had evidence of a pancreatic mass within the residual left pancreatic lobe and possible lymphadenopathy on follow-up abdominal imaging. At the time of study completion, 3/6 dogs were alive, 2/6 dogs were dead, and 1/6 dog was lost to follow-up at 400 days postoperatively. Two dogs (Dogs 3 and 4) were euthanized at 155 days and 177 days following surgery due to progression of insulinoma and clinical signs associated with hypoglycemia despite treatment. The three dogs known to be alive at the time of study completion had a median follow-up time of 383 days (range 294–1080).

## Discussion

Based on the results of this trial, targeted NIR imaging utilizing a cathepsin-activated fluorophore warrants further investigation for intraoperative detection of canine insulinoma. VGT-309, when administered via the described dose and route, appears to be safe with no adverse events related to administration in any dog. It was effective in identifying pancreatic insulinomas; all dogs demonstrated enhanced mean fluorescence intensity of the insulinoma relative to the surrounding pancreas, and fluorescence margins correlated well with histologic margins. This included one dog in which a pancreatic mass was not accurately identified on preoperative imaging (ultrasound alone) or initial abdominal exploration. The mean TBR was 1.906 (range 1.286–2.556). Two dogs had TBRs < 1.5. These two insulinomas were clearly visible in both dogs using NIR fluorescence imaging during surgery in spite of the low TBR. One study evaluating signal to background ratio found that a ratio of at least 1.5 is needed to reliably discriminate fluorescent lesions from surrounding tissues [39]. However canine mammary and primary lung tumors with TBRs < 1.5 have been identified using intraoperative NIR fluorescence [34,36]. Intraoperative detection of neoplasms using NIR fluorescence will vary depending on the tumor type, the imaging agent used and dose administered, and the imaging system employed. The lower TBR limit of canine insulinoma detection with the current cathepsin-activated NIR imaging agent and system employed is unclear; however the results of this study suggest that this protocol may aid in primary tumor detection and margin evaluation.

Cathepsin B IHC results were consistent with the intraoperative NIR imaging findings, as insulinomas demonstrated greater cathepsin B IHC positivity compared to the surrounding non-neoplastic pancreas. There was no overlap in IHC-positivity proportion between the non-neoplastic pancreas and insulinoma samples in these dogs. There was not a direct correlation between the ratios of mean fluorescence intensity and IHC (TBR and IHCr, respectively) seen in insulinomas and adjacent non-neoplastic pancreatic tissues. For instance, Dog 1 had the greatest IHCr but the second lowest TBR. Importantly, the imaging agent utilized in this study is an activity-based, cathepsin-agnostic (i.e., binds to all cathepsins) probe, whereas the IHC antibody used is specific for cathepsin B. Future validation and use of global cathepsin antibodies for IHC are warranted to further assess the correlation between mean fluorescence intensity with VGT-309 and IHC. In addition, incorporation of fluorescence microscopy may also be beneficial in assessing the relation of these findings. There was overlap in the range of IHC-positivity proportion values for insulinoma samples and non-neoplastic lymph node and liver tissues. Both the liver and lymph nodes have endogenous cathepsin expression, which accounts for the fluorescence identified intraoperatively in these tissues.

Background fluorescence was identified in the liver and lymph nodes of all dogs. Cells within both these tissue types seemingly express cathepsins capable of activating the fluorophore used in this study. It was not possible to definitively discern metastatic lesions within these tissues on the basis of fluorescence. For the one dog with stage 3 insulinoma, there was no appreciable difference in fluorescence of metastatic nodules within the liver relative to surrounding liver tissue, and a large, effaced, metastatic lymph node had a lower fluorescence intensity compared to a concurrently excised, non-metastatic lymph node. The variable nature of fluorescence intensity of the lymph nodes relative to the insulinomas in other cases without identified nodal metastasis, and the finding of a hyperfluorescent but benign liver nodule relative to surrounding liver, underscore the need for further investigation of NIR imaging on intraoperative lymph node and liver assessment in these patients. Altered protocols utilizing cathepsin-activated fluorophores, such as dosing and timing of injection relative to surgery, should be investigated. These strategies may increase washout of the agent from non-neoplastic tissues that have native cathepsin levels but enhanced lymphatic drainage (relative to neoplastic tissues) in an effort to improve identification of metastatic lesions while still demonstrating selectively enhanced uptake and retention within the primary pancreatic tumor. Based on preclinical mouse models with VGT-309, washout from non-neoplastic structures is anticipated to be improved with a greater interval between injection and imaging [28]. For dogs in a prior lung tumor study, this agent was generally administered on the same day as surgery [28]. However, for humans with lung tumors receiving VGT-309 in this study, the imaging agent was administered 1 day preoperatively [28]. Therefore, an injection interval of 1 day preoperatively was initially chosen for dogs of the present study. Increasing the interval between agent administration and surgery may conceivably allow washout from normal tissues with high native cathepsin levels, resulting in less fluorescence than neoplastic tissues with high cathepsin levels due to enhanced retention and altered lymphatics within the tumor tissues. An IACUC amendment allowed for this in one dog, and further modification of injection interval is needed to evaluate this effect on NIR findings. However, investigation of other targeted imaging agents that are highly selective for insulinoma tumor-bearing tissues is also needed. In light of research on somatostatin receptor-targeted PET imaging for human benign and malignant insulinoma, further investigation of a targeted somatostatin receptor-based NIR imaging agent should be considered for evaluation in dogs and humans with this disease. Ultimately, accurate intraoperative identification of the primary tumor and all metastatic lesions is crucial to appropriately staging disease and improving patient outcomes by allowing for enhanced cytoreduction of disease. Development of targeted NIR agents and protocols that are safe, sensitive, and specific for insulinoma may play an important role in improving intraoperative disease localization.

There were several limitations of this study. First, the sample size was small with an even smaller subgroup of patients with documented metastatic disease at the time of surgery. This limited the ability to evaluate for differences in intraoperative NIR findings for patients with vs. without metastatic disease, as well as for metastatic vs. nonmetastatic lesions within patients with metastatic disease. A larger sample size will be needed to further characterize these findings, as well as to statistically evaluate for differential fluorescence intensity between metastatic and nonmetastatic lesions. Given the sample size limitations, definitive conclusions on efficacy, sensitivity and specificity, and impact on surgical or patient outcomes cannot be made. In addition, this study was exploratory in nature and has provided important pilot data, but as such, it is possible that alteration of protocols relative to administration of the cathepsin-activated imaging agent could yield different results. Furthermore, advanced preoperative imaging modalities such as CT were offered but not required for all dogs in this study, and in cases in which intraoperative findings differed from preoperative non-CT based imaging (such as Dog 4) it is unknown whether CT would find similar results relative to surgery. Moreover, gain settings were not standardized for each case and were adjusted relative to intraoperative findings and tissues being evaluated; all gain settings have been provided relative to fluorescence quantification. Standardization of gain settings throughout cases in future studies will be valuable in providing direct comparisons relative to fluorescence quantification across all patients and tissue types. In addition, standardization of anesthetic and glycemic management should be considered in future studies to allow for assessment of blood glucose changes relative to NIR findings and excision of lesions intraoperatively. Two dogs in this study were managed with steroids preoperatively, which has the potential to affect cathepsin expression [40]. The effect of preoperative prednisone administration on cathepsin-based NIR findings in these dogs is not known, as one of these dogs had TBR < 1.5 and the other had TBR > 1.5. Future studies should take this into account and attempt to standardize preoperative management that could potentially alter targeted NIR results. Use of fluorescence microscopy to localize the transition in fluorescence intensity on a cellular level would be beneficial and should be incorporated into future studies. Finally, the effect of this intraoperative imaging technique on patient and oncologic outcomes remains to be studied. To adequately assess this, a larger sample size and standardization of follow-up and adjuvant therapy administration will be needed.

A future study would first need to optimize NIR conditions, including determination of optimal gain setting, imaging agent dose, and duration between injection and imaging, and also theorize an expected correlation coefficient (r) relative to the chosen variable for correlative assessment (i.e., preoperative CT findings, intraoperative gross findings, or histopathology results). Variables to assess, once an optimized imaging agent and a properly powered study are designed, include: negative margin rates, identification of occult synchronous lesions, accurate localization of the tumor and metastatic disease, glycemic control postoperatively, times to disease progression, and survival times. As this disease is relatively rare in veterinary medicine, initiation of a multi-institutional study is likely to be needed to obtain adequate sample size (based on the power analysis) within a reasonable timeframe.

In conclusion, this prospective study evaluated the use of intraoperative targeted NIR imaging with a cathepsin-activated fluorophore in dogs with insulinoma. The findings of this pilot study support further investigation of this imaging agent and technique for intraoperative disease localization. Further studies on altered administration protocols of a cathepsin-activated imaging agent as well as alternative selective imaging agents are needed to enhance intraoperative metastatic disease identification and maximize sensitivity and selectivity of targeted NIR imaging for canine insulinoma. Future controlled studies including a large number of dogs undergoing targeted NIR imaging will be important to determine effect on patient outcomes, and dogs with insulinoma may serve as an effective translational model for targeted NIR imaging in humans with pancreatic neuroendocrine tumors.

## Supporting information

S1 File **Fig. S1.** Formalin-fixed paraffin embedded slide (1.6x) of non-metastatic liver in Dog 6 stained with cathepsin B antibody for IHC analysis; the scale bar represents 2 mm. **Fig. S2.** Formalin-fixed paraffin embedded slide (1.6x) of non-metastatic splenic lymph node (cortex) in Dog 6 stained with cathepsin B antibody for IHC analysis; the scale bar represents 2 mm. **Fig. S3.** Western blot cathepsin B antibody validation. The single band of appropriate molecular weight (~30 kDa) demonstrates specificity and cross-reactivity in both human cell lines (MG63, SK-MEL-28) and canine cell lines (Abrams, STSA1). **Table S1.** Thresholding parameters for the positive pixel count algorithm in ImageScope.
(DOCX)

S1 Appendix IHC protocol. (DOCX)

S2 Appendix Western blot protocol. (DOCX)

Supplemental Data Image J and IHC data for all dogs. (XLSX)
