# Supplementary material for: Targeted near-infrared imaging utilizing a cathepsin-activated fluorophore for the intraoperative detection of canine insulinoma
Source: PLoS One. 2026 Feb 23;21(2):e0343299. doi: 10.1371/journal.pone.0343299 (PMC12928447; doi:10.1371/journal.pone.0343299)
Supplement: S1 File — Fig. S1. Formalin-fixed paraffin embedded slide (1.6x) of non-metastatic liver in Dog 6 stained with cathepsin B antibody for IHC analysis; the scale bar represents 2 mm. Fig. S2. Formalin-fixed paraffin embedded slide (1.6x) of non-metastatic splenic lymph node (cortex) in Dog 6 stained with cathepsin B antibody for IHC analysis; the scale bar represents 2 mm. Fig. S3. Western blot cathepsin B antibody validation. The single band of appropriate molecular weight (~30 kDa) demonstrates specificity and cross-reactivity in both human cell lines (MG63, SK-MEL-28) and canine cell lines (Abrams, STSA1). Table S1. Thresholding parameters for the positive pixel count algorithm in ImageScope. (DOCX) [file pone.0343299.s001.docx]

**Table S1:** Thresholding parameters for the positive pixel count algorithm in ImageScope

| Pixel Area (millimeter-squared) | 7.54562e-008 |
| --- | --- |
| Hue Value (Center) | 0.1 |
| Hue Width | 0.5 |
| Color Saturation Threshold | 8.e-002 |
| Intensity Threshold WEAK (Upper Limit) | 200 |
| Intensity Threshold WEAK (Lower Limit) | 175 |
| Intensity Threshold MEDIUM (Upper Limit) | 175 |
| Intensity Threshold MEDIUM (Lower Limit) | 100 |
| Intensity Threshold STRONG (Upper Limit) | 100 |
| Intensity Threshold STRONG (Lower Limit) | 0 |
| Intensity Threshold Negative Pixels | 253 |

**Fig S1:** Formalin-fixed paraffin embedded slide (1.6x) of non-metastatic liver in Dog 6 stained with cathepsin B antibody for IHC analysis; the scale bar represents 2 mm.

**Fig S2:** Formalin-fixed paraffin embedded slide (1.6x) of non-metastatic splenic lymph node (cortex) in Dog 6 stained with cathepsin B antibody for IHC analysis; the scale bar represents 2 mm.

**Fig S3:** Western blot cathepsin B antibody validation. The single band of appropriate molecular weight (~30 kDa) demonstrate specificity and cross-reactivity in both human cell lines (MG63, SK-MEL-28) and canine cell lines (Abrams, STSA1).
