# Supplementary material for: Targeted near-infrared imaging utilizing a cathepsin-activated fluorophore for the intraoperative detection of canine insulinoma
Source: PLoS One. 2026 Feb 23;21(2):e0343299. doi: 10.1371/journal.pone.0343299 (PMC12928447; doi:10.1371/journal.pone.0343299)
Supplement: S1 Appendix — (DOCX) [file pone.0343299.s002.docx]

**Antibody:** Cathepsin B, CST #31718, Rb mAb.

**Application:** IHCp.

**Species reactivity:** Can, Ms (other species not tested).

**Protocol:**

For immunohistochemistry on formalin fixed paraffin embedded material, 5 µm thick paraffin sections mounted on ProbeOn™ slides (Thermo Fisher Scientific).

The immunostaining procedure is performed using a Leica BOND RX^m^ automated platform combined with the Bond Polymer Refine Detection kit (Leica DS9800) and includes the following steps:

1. After dewaxing (Leica AR9222) and rehydration in dH2O, sections are pretreated with the epitope retrieval BOND ER2 high pH buffer (EDTA Based pH=9.0, Leica AR9640) (20 min, 95°C).
2. Endogenous peroxidase is inactivated with 3% H2O2 (10 min, RT).
3. Nonspecific protein-protein interactions are blocked with Leica PowerVision IHC/ISH Super Blocking solution (Leica PV6122) for 30 minutes at RT.
4. The primary antibody is diluted 1/600 using the Cell Signaling Technology (CST) diluent solution and applied for 45 minutes at RT.
5. A biotin-free polymeric IHC detection system (Leica DS9800) consisting of HRP conjugated Gt anti-Rb IgG secondary antibody is applied for 25 minutes at RT.
6. Immunoreactivity is revealed with the diaminobenzidine (DAB) chromogen reaction.

Slides are finally counterstained in hematoxylin, dehydrated in an ethanol series, cleared in xylene, and permanently mounted with a resinous mounting medium (Thermo Scientific ClearVueTM coverslipper).

TBS (Leica AR9590) is used as a washing solution in between steps.

Positive control: normal pancreas.

Negative control: irrelevant isotype-matched Rb mAb.

**Abbreviation List**

Can-Canine, Gt-Goat, Ms-Mouse, Rb-Rabbit.

mAb-Monoclonal Antibody.

RT-Room Temperature.

IHCp-Immunohistochemistry on Paraffin Sections.
