# Supplementary material for: Targeted near-infrared imaging utilizing a cathepsin-activated fluorophore for the intraoperative detection of canine insulinoma
Source: PLoS One. 2026 Feb 23;21(2):e0343299. doi: 10.1371/journal.pone.0343299 (PMC12928447; doi:10.1371/journal.pone.0343299)
Supplement: S2 Appendix — (DOCX) [file pone.0343299.s003.docx]

*Western Blot*

Cell lines were grown in Dulbecco’s modified Eagle medium (DMEM) supplemented with 10% fetal bovine serum, MEM Vitamin solution, 2 mM L-glutamine, 1 mM sodium pyruvate, non-essential amino acids, and antibiotic/antimycotic solution (Corning, Corning, NY). All cells were grown in a 37°C humidified atmosphere of 5% CO_2_ and serially passaged by trypsinization. When cells reached 70% confluency, the flasks were scraped, cells pelleted, then lysed with cell lysis buffer [7 mL M-Per (Thermo Scientific, Waltham, MA), 140 mg sodium dodecyl sulfate (Fisher Chemical, Waltham, MA), 1/2 Complete Mini Protease Inhibitor Cocktail tablet (Roche, Indianapolis, IN), 1 mM NaOVA (Sigma, St. Louis, MO), 1 mM PMSF (Sigma, St. Louis, MO)] on ice for 5 minutes. Lysates were then aspirated using a 25G needle 5x, centrifuged at 18000 x g for 10 minutes at 4°C, and supernatants collected for western blot. A BCA assay (Biorad, Hercules, CA) was performed to quantify protein concentrations and equal protein amounts were boiled for 5 min with SDS sample buffer. Proteins were separated using SDS-PAGE on a NuPAGE 4–12% Bis-Tris protein gel (Invitrogen, Waltham, MA) followed by transfer onto PVDF membranes (Immobilon- Milipore, Burlington, MA) and blocking with Superblock T20 (TBS) Blocking Buffer (Thermo Scientific, Waltham, MA) for 1 hour at room temperature. Primary antibodies [anti-Cathepsin B (Cell Signaling #D1C7Y, Cell Signaling, Danvers, MA), anti-B-Actin (Novus Bio #NB600-503, Novus Biologicals, Centennial, CO)] were used at 1:1000 dilutions in Superblock T20 (TBS) Blocking Buffer at 4°C overnight. Membranes were washed 3x with TBST before incubation with secondary antibody at 1:40,000 (Goat anti-rabbit IgG HRP-conjugated (Thermo Scientific Waltham, MA)) for 1 hour at room temperature followed by another 3x wash with TBST. Chemiluminescent substrate (Supersignal West Pico, Thermo Scientific Waltham, MA) was then applied to membranes and imaged using a Chemi Doc XES+ imaging system (BioRad, Hercules, CA).
